# Identification and Description of Regulatory and Health Technology Assessment Agencies’ Guidance Related to Patient Experience Data in North America, Europe, and Asia Pacific

**DOI:** 10.1007/s43441-025-00777-z

**Published:** 2025-07-17

**Authors:** Laure Delbecque, Juergen Zschocke, Ding Ding, Jiat-Ling Poon, Carolina Alonzo, Nancy Gabriela Pérez, Shane Myrick, Jennifer N. Hill

**Affiliations:** 1 Eli Lilly and Company, Indianapolis, IN USA; 2 IQVIA, New York, NY USA

**Keywords:** Clinical outcome assessment, Health technology assessment, Patient experience data, Patient-reported outcome, Regulatory

## Abstract

**Objectives:**

To identify and describe patient experience data (PED)-related guidance issued by regulatory and health technology assessment (HTA) agencies in North America, Europe, and Asia Pacific.

**Methods:**

National agency websites were manually searched (October 2021, updated October–December 2023) to identify standalone PED guidance, and general submission/clinical trial or disease-specific guidance in four therapy areas, including PED-related recommendations. Identified documents were reviewed for requirements/expectations on types of PED; concepts of interest and instruments/tools; validation, analysis, and interpretation; study design, and endpoint definitions.

**Results:**

A total of 34 regulatory and 21 HTA documents were reviewed across 7 and 11 agencies, respectively. Most regulatory agencies expected data collected via clinical outcome assessments; concepts of interest were similar; specific tools were not usually recommended, although the use of validated instruments was expected; and meaningful within-patient change was generally considered key to interpretation. HTA guidance on PED varied primarily based on country requirement for utility estimates to inform economic evaluation (little/no guidance beyond the concept of interest) versus interest in PED on signs, symptoms, or disease impact on functioning (generally more comprehensive details on PED expectations).

**Conclusion:**

Through PED, patient voice is an increasingly important factor in regulatory, access, and reimbursement decision-making. Regulatory and HTA agencies in North America, Europe, and Asia Pacific have varying expectations regarding PED. This misalignment can necessitate the collection of multiple types of PED to meet all requirements (resulting in complex clinical trial protocols and significant patient burden), and lead to differences in data interpretation or gaps in the expected data. Therefore, PED measurement standards should be harmonized globally, and the collection of required PED should be ensured early during drug development to ensure decisions can be made based on valid and reliable data.

## Introduction

Successfully bringing a new medicine to market is dependent on achieving market authorizations from regulatory agencies and positive reimbursement decisions, often based on health technology assessments (HTAs) [1]. While regulatory agencies focus on risk–benefit assessments under ideal circumstances, largely based on clinical trial data [2], HTA agencies and HTA organizations (hereafter described collectively as HTA agencies) consider the overall value of new medicines, sometimes evaluating their relative cost effectiveness compared with existing treatment(s) [3]. Given their different roles, regulatory and HTA agencies may have different evidentiary requirements for determining the benefit–risk and value of treatments, respectively.

Clinical trials are often long, costly, and burdensome for patients [4, 5]; therefore, it is important that they are designed to meet the data requirements of all stakeholders involved in healthcare (i.e., patients, healthcare professionals [HCPs], regulators, health technology assessors, and payers), allowing them to make informed choices about new treatments. Considering and incorporating all stakeholder objectives and evidentiary needs early in the drug development process should minimize the need for additional evidence generation. This approach should also avoid situations in which new medicines achieve regulatory approval but fail to be reimbursed due to a lack of evidence for HTA, or achieve regulatory approval and reimbursement but have limited uptake because they do not fulfill patients’ unmet needs (e.g., drugs with limited therapeutic innovation, unknown long-term risks, or complex administration).

In addition to clinical data, patient experience data (PED) are increasingly being used by regulatory and HTA agencies to capture patient-centered dimensions and support their review and decision-making processes [2], thereby informing approval and reimbursement decisions, respectively, and impacting access to new treatments [6, 7]. PED offer insights into patients’ experiences of a disease and/or with a treatment; therefore, PED are key to ensuring that patients’ medical needs are met by new treatments. There is currently no standard definition of PED, but the United States (US) Food and Drug Administration (FDA) advises that “PED can be interpreted as information that captures patients’ experiences, perspectives, needs, and priorities related to (but not limited to): (1) the symptoms of their condition and its natural history; (2) the impact of conditions on their functioning and quality of life (QoL); (3) their experience with treatment; (4) input on which outcomes are important for them; (5) patient preferences for outcomes and treatments; and (6) the relative importance of any issues as defined by patients” [8]. In addition, PED may capture other aspects of healthcare experiences, such as patient satisfaction, treatment adherence, and perceptions of clinical trials, healthcare systems, and/or procedures [9]. PED can be generated using several methods, including clinical outcome assessments (COAs), which assess how patients feel, function, or survive; preference studies; qualitative research; or natural history examinations.

There is currently no standardized or globally recognized process for capturing, analyzing, reporting, or reviewing PED and incorporating it in regulatory and/or HTA decision-making processes, and each agency independently decides whether to develop its own PED guidance. As a result, differing considerations and approaches to PED can add complexity to the drug development process and have the potential to lead to different approval and access decisions for new medicines. This is particularly important given the trend towards a broader range of countries being included in multinational clinical trials and leveraging the same evidence for market access [10], potentially depriving some patients of beneficial new treatments.

The objective of this study was to identify and describe guidance relating to PED issued by regulatory and HTA agencies in North America, Europe, and Asia Pacific. Where relevant guidance documents were identified, we examined the inclusion of PED recommendations related to COAs or other forms of PED, as well as the types of evidence that regulatory and HTA agencies expect to see.

## Materials and Methods

### Scope

National regulatory and HTA agencies across the geographic regions within the scope of this analysis are outlined in Table 1. In the US, the Institute for Clinical and Economic Review (ICER), an independent non-profit research organization that evaluates medical evidence, convenes public deliberative bodies to help stakeholders interpret and apply evidence to improve patient outcomes and control costs. ICER provides specific guidance regarding HTA submissions, which are widely used by stakeholders, including regulatory bodies such as the FDA and US payers [11–13]. ICER was selected for inclusion for this analysis due to its guidance being extensively utilized across these key stakeholder groups [11, 14, 15]. Given that US payers (e.g., Humana, United Healthcare, Aetna, Cigna, Anthem, and Medicare) often use ICER’s guidance to inform their coverage and reimbursement decisions [12, 13], and their remits differ distinctly from those of an HTA agency, they were excluded from this analysis. In Europe, national regulatory agencies were not included, as treatments in the four therapy areas of interest (diabetes, inflammatory autoimmune diseases, neurodegenerative disease, and oncology) are eligible for the European Medicines Agency (EMA) centralized procedure [16]. As there is no official national HTA agency in China, the National Healthcare Security Administration, the national reimbursement decision-making agency [17], and the National Center for Medicine and Health Technology Assessment (NCMHTA), an affiliated research institution [18], were selected for inclusion in this analysis. Regional regulatory and HTA agencies were excluded.

**Table 1:** Geographies/Countries and Respective Regulatory and Health Technology Assessment Agencies/Organizations within the Scope of this Analysis

Geography/country	Regulatory agency	Health technology assessment agency/organization
*North America*		
Canada	Health Canada	Canadian Agency for Drugs and Technologies in Health
United States	Food and Drug Administration	Institute for Clinical and Economic Review
*Europe*^*a*^		European Network for Health Technology Assessment
France	European Medicines Agency	Haute Autorité de Santé
Germany	European Medicines Agency	Federal Joint Committee Institute for Quality and Efficiency in Health Care
Italy	European Medicines Agency	Agenzia Italiana del Farmaco
Spain	European Medicines Agency	Inter-Ministerial Pricing Commission for Pharmaceuticals Grupo de Evaluación de Novedades, Estandarización e Investigación en Selección de Medicamentos Spanish Agency of Medicines and Medical Devices
United Kingdom	Medicines and Healthcare products Regulatory Agency	National Institute for Health and Care Excellence
*Asia–Pacific*		
Australia	Therapeutic Goods Administration	Pharmaceutical Benefits Advisory Committee
China	National Medical Products Administration Center for Drug Evaluation	National Healthcare Security Administration National Center for Medical and Health Technology Assessment
Japan	Pharmaceuticals and Medical Devices Agency	Center for Outcomes Research and Economic Evaluation for Health

^a^The European Medicines Agency is a decentralized body of the European Union and represents the competent authorities of (amongst others) France, Germany, Italy, and Spain

### Guidance Document Searches

Each regulatory and HTA agency website was initially searched in October 2021 using a manual process (“hand search”) to identify standalone guidance documents exclusively on how PED should be included in regulatory or HTA submissions, respectively, published by the agency. Handsearching—using keyword searches to locate potentially relevant documents that are examined by researchers to determine their suitability for inclusion—is a practice that is recommended when highly structured searches (e.g., those on traditional websites) are not possible, and when page-by-page searches of websites are required to review and identify sources [19]. Terms for the website searches included outcomes of interest: “COA”, “PED”, “patient perspective(s)”, “patient preference”, “patient-reported outcome(s)” (PRO[s]), and “QoL”. Search terms were translated into the local language by project team members using online translation tools when the website under review did not publish English versions of guidance documents. The majority of regulatory and HTA agencies within scope encompass both pharmaceuticals and medical devices; however, documents related only to medical devices or therapeutic areas not within the scope of this work were excluded. Documents describing patient engagement activities (i.e., the engagement of patients in the regulatory or HTA process) were excluded to focus searches specifically on PED.

If no published standalone guidance on PED was identified, general guidance on regulatory submissions, clinical trial development, and/or HTA submissions were searched in October 2021 and reviewed for PED-related information using the same outcome search terms.

A third search of each regulatory and HTA agency website was conducted in October 2021 to identify published disease-specific guidance that included recommendations on the generation and use of PED in four therapy areas of interest: diabetes (specifically type 2 diabetes mellitus [T2DM]), inflammatory autoimmune diseases (specifically atopic dermatitis, systemic lupus erythematosus [SLE], and ulcerative colitis), neurodegenerative disease (specifically Alzheimer’s disease), and oncology (prioritizing female breast cancer and non-small cell lung cancer [NSCLC], if available). Disease-specific search terms were “diabetes type 2”, “T2DM”, “atopic dermatitis”, “SLE”, “ulcerative colitis”, “Alzheimer’s disease”, “oncology”, “breast cancer”, and “NSCLC”. The outcome of interest terms for the disease-specific searches were as described above.

The three initial searches conducted in October 2021 were repeated between October and December 2023 to determine whether draft guidance identified in the initial searches had been updated, and/or whether any new guidance documents were issued between 2021 and 2023. Where draft guidance documents were updated, only findings from the updated guidance documents are reported.

### Guidance Document Reviews

A detailed review of each identified guidance was undertaken to obtain information related to: (1) types of PED evidence required or expected by regulatory or HTA agencies (e.g., COA, preference study/survey, qualitative research, or natural history examination); (2) expectations regarding concepts of interest (e.g., signs, symptoms, health-related quality of life [HRQoL], and utilities) and instruments/tools (i.e., generic, or disease-specific); (3) requirements for COA validation, analysis, and interpretation; (4) requirements for study design elements (including handling of missing data); and (5) considerations for endpoint definitions. Initial document reviews were conducted by individual project team members, and all results were reviewed by the principal project lead. When interpretation disagreements or questions arose, the project team members, principal project lead, and project scientific lead met to address those points. Where there was limited or no information available for an agency, this was highlighted.

## Results

### Regulatory Agency Guidance on PED

Overall, 34 unique guidance documents were reviewed across seven regulatory agencies in North America, Europe, and Asia Pacific. Thirty-two guidance documents were identified in the initial 2021 search. Between 2021 and 2023, five draft guidance documents were updated, one guidance document was withdrawn, and three new guidance documents were issued. Table 2 summarizes all guidance documents that were reviewed (i.e., the most recent versions still accessible on relevant agency websites in 2023).

**Table 2:** Regulatory Agency Guidance Documents Reviewed by Agency and Geography/Country

Document type identified yes/no n, (publication year(s))	HC	FDA	EMA	MHRA	TGA	NMPA-CDE	PMDA
Standalone guidance on PED^a^	No	Yes n = 7 [20–26]	Yes n = 2 [27, 28]	No	Yes^d^ n = 1 [27, 29]	Yes n = 4 [30–33]	No
General guidance document(s)^b^ including PED-related recommendations	No	N/A	N/A	No	N/A	N/A	n = 1 [34]
Disease-specific guidance document(s)^c^ including PED-related recommendations	No	Yes	Yes	No	No	Yes	Yes
Diabetes (specifically T2DM)	–	n = 1 [35]	n = 1 [36]	–	–	n = 2 [37, 38]	n = 1 [39]
Inflammatory autoimmune disorders (specifically atopic dermatitis, SLE, and UC)	–	n = 3 [40–42]	n = 2 [43, 44]	–	–	n = 1 [45]	–
Neurodegenerative disease (specifically Alzheimer’s disease)	–	n = 1 [46]	n = 1 [47]	–	–	–	n = 1 [48]
Oncology (specifically breast cancer and NSCLC, if available)	–	n = 3 [49–51]	n = 1 [52]	–	–	n = 1 [53]	n = 1 [54]

^a^Standalone guidance document(s) exclusively about PED

^b^General guidance document(s) on regulatory submissions and/or clinical trial development. If standalone guidance on PED was found, general guidance document(s) including PED-related recommendations were not reviewed (i.e., search not applicable)

^c^Disease-specific guidance document(s) in four therapy areas of interest

^d^TGA adopted EMA standalone guidance on PED related to oncology only (Appendix 2 to the guideline on the evaluation of anticancer medicinal products in man. The use of patient-reported outcome (PRO) measures in oncology studies. April 2016) in 2023

EMA, European Medicines Agency; FDA, Food and Drug Administration; HC, Health Canada; MHRA, Medicines and Healthcare products Regulatory Agency; N/A, not applicable; NMPA-CDE, National Medical Products Administration Center for Drug Evaluation; NSCLC, non-small cell lung cancer; PED, patient experience data; PMDA, Pharmaceuticals and Medical Devices Agency; SLE, systemic lupus erythematosus; T2DM, type 2 diabetes mellitus; TGA, Therapeutic Goods Administration; UC, ulcerative colitis

A total of 13 standalone guidance documents on PED were reviewed: seven issued by the US FDA, two by the EMA, and four by the China National Medical Products Administration Center for Drug Evaluation (NMPA-CDE). In addition, the Australian regulatory agency (Therapeutic Goods Administration [TGA]) adopted a series of international guidance in 2023, including the EMA 2016 “Appendix 2 to the guideline on the evaluation of anticancer medicinal products in man: the use of PRO measures in oncology studies” [27] to ensure harmonization of data requirements with those of other international regulators. No standalone guidance on PED has been issued by the Canadian, United Kingdom (UK), or Japanese regulatory agencies. One instance of PED-related recommendations being included in a general guidance document on regulatory submissions and/or clinical trial development issued by the Pharmaceuticals and Medical Devices Agency (PMDA) in Japan was identified. Overall, 20 disease-specific guidance documents that included PED-related recommendations were issued by regulatory agencies in therapy areas of interest in the US (n = 8), Europe (n = 5), China (n = 4), and Japan (n = 3). The Canadian and UK agencies, Health Canada and the Medicines and Healthcare products Regulatory Agency (MHRA), respectively, were the only regulatory agencies for which no PED-related guidance or recommendation was identified.

Across regulatory agencies that discussed PED, the data were expected to be collected through COA in most cases; PED-related concepts of interest were similar (i.e., signs, symptoms, impact, functioning, and HRQoL); and none of the agencies recommended specific tools, except in some disease-specific PED-related guidelines (Table 3). The FDA and China’s NMPA-CDE were open to more different types of PED, including social media and qualitative studies. Recommendations for the use of validated instruments were also consistent across agencies, but the US FDA was much more detailed about its validation expectations, providing suggestions for how to validate tools, whereas the other agencies were vague beyond the requirement to use validated instruments. A double-blind study design was broadly recommended to avoid bias; COA were considered acceptable primary, co-primary, secondary, and exploratory endpoints; and expectations regarding missing data were documented. Meaningful within-patient change (MWPC) was generally considered key to COA interpretation; however, the EMA, and by extension Australia’s TGA, which adopted EMA guidance, were also interested in group difference. Overall, the FDA provided the most detailed PED-related guidance, whereas Japan’s PMDA provided the least detailed guidance**.**

**Table 3:** Overview of Regulatory Agency Recommendations Regarding Patient Experience Data by Agency and Geography/Country

	FDA	EMA	TGA^a^	NMPA-CDE	PMDA
Types of PED	COAs, qualitative studies, surveys, preference studies^b^	COAs, qualitative and quantitative studies	COAs	COAs, qualitative studies, social media, surveys	COAs
Concepts of interest	Signs, symptoms, disease impact on HRQoL, functioning, treatment satisfaction	Symptoms, disease impact on HRQoL, functioning, treatment satisfaction and adherence	Symptoms, disease impact on HRQoL, functioning, treatment satisfaction and adherence	Signs, symptoms, disease impact on HRQoL, functioning, preference, treatment satisfaction, and tolerability	“Patient benefits”
Recommended instruments/tools	No specific recommendation about instruments but some disease-specific guidance documents mention specific tools for sponsor consideration	No specific recommendation about instrument but some disease-specific guidance documents mention specific tools for sponsor consideration	No specific recommendation about instrument but some disease-specific guidance documents mention specific tools for sponsor consideration	No specific recommendation about instruments	No specific recommendation about instruments but some disease-specific guidance documents mention specific tools for sponsor consideration
COA validation	Provide specific guidelines on how to validate an instrument and specify which evidence should be communicated to the agency to support the use of the instrument	Recommend the use of validated instruments (with acceptable responsiveness, reliability, and validity) and expect evidence of instrument validation (and of cultural adaptation/translation if applicable)	Recommend the use of validated instruments (with acceptable responsiveness, reliability, and validity) and expect evidence of instrument validation (and of cultural adaptation/translation if applicable)	Recommend the use of validated instrument and specify which evidence should be communicated to the agency to support the use of the instrument. Language and cultural validation on the local population should be considered	Recommend the use of instrument developed and validated per “scientific methods.” Language and cultural validation on the local population should be considered
COA analyses/interpretation	MWPC is key; threshold defined from anchor-based analyses	MWPC (magnitude of relevance of change based on relevant patient-based and clinical anchors) and between-group mean difference	MWPC (magnitude of relevance of change based on relevant patient-based and clinical anchors) and between-group mean difference	MWPC is key; thresholds based on expert consensus, guidelines, and PED	Consider clinical significance of assessment using scale
Missing data^c^	Should be justified and addressed	Should be justified and addressed	Should be justified and addressed	Should be justified and addressed	N/A
Trial design	Provide recommendations on designing and implementing COA assessments in clinical trials. COAs are acceptable primary (although rarely), co-primary, secondary, and exploratory endpoints	Provide recommendations on designing and implementing COA assessments in clinical trials. COAs are acceptable primary (although rarely), co-primary, secondary, and exploratory endpoints	Provide recommendations on designing and implementing COA assessments in oncology clinical trials. COAs are acceptable primary (although rarely), secondary, and exploratory endpoints	Provide recommendations on designing patient-centered clinical trials	N/A
Endpoints	Full guidance [26] developed on selecting the COA-based endpoint and determining clinically meaningful change in that endpoint	COA endpoints should be meaningful to patients	COA endpoints should be meaningful to patients	COAs are acceptable endpoints	COAs to support efficacy and safety endpoints

No HC or MHRA guidance documents were identified; therefore, these agencies are excluded from this agency recommendations table

^a^TGA adopted EMA standalone guidance on PED related to oncology only (Appendix 2 to the guideline on the evaluation of anticancer medicinal products in man. The use of patient-reported outcome (PRO) measures in oncology studies. April 2016) in 2023

^b^Specific guidance on patient preference information provided

^c^Guidance on acceptable levels of missing data and how best to handle missing data during analyses

COA, clinical outcome assessment; CTCAE, Common Terminology Criteria for Adverse Events; EMA; European Medicines Agency; EORTC, European Organisation for Research and Treatment of Cancer; FACT, Functional Assessment of Cancer Therapy; FDA, Food and Drug Administration; HC, Health Canada; HRQoL, health-related quality of life; MHRA, Medicines and Healthcare products Regulatory Agency; MWPC, meaningful within-patient change; N/A, not applicable; NMPA-CDE, National Medical Products Administration Center for Drug Evaluation; PED, patient experience data; PMDA, Pharmaceuticals and Medical Devices Agency; PRO, patient-reported outcome; TGA, Therapeutic Goods Administration

### HTA Agency Guidance on PED

Overall, 21 guidance documents across 11 HTA agencies in North America, Europe, and Asia Pacific were identified and reviewed (Table 4). Twelve guidance documents were identified in the initial 2021 search, and nine new guidance documents were issued between 2021 and 2023.

**Table 4:** Health Technology Assessment Agency/Organization Guidance Documents Identified by Agency/Organization and Geography/Country

Document type identified yes/no; n	CADTH	ICER	EUnetHTA	HAS	G-BA & IQWiG	AIFA	CIPM, GENESIS & AEMPS	NICE	PBAC	NHSA & NCMHTA	C2H
Standalone guidance on PED^a^	No	No	Yes n = 2^d^ [55, 56]	No	No	No	No	No	No	No	No
General guidance document(s)^b^ including PED-related recommendations	Yes n = 3 [57–59]	Yes n = 1 [60]	Yes n = 1 [61]	Yes n = 2 [62, 63]	Yes n = 2 [64, 65]	Yes n = 1 [66]	Yes n = 1 [67]	Yes n = 3 [68–70]	Yes n = 1 [71]	No	Yes n = 1 [72]
Disease-specific guidance document(s)^c^ including PED-related recommendations	Yes	No	No	No	No	No	No	No	No	Yes	No
Inflammatory autoimmune disorders (specifically atopic dermatitis, SLE, and UC)	n = 1 [73]										
Oncology (specifically breast cancer and NSCLC)	n = 1 [74]	–	–	–	–	–	–	–	–	n = 1 [75]	–

^a^Standalone guidance document(s) exclusively about PED

^b^General guidance document(s) on HTA submissions and/or clinical trial development

^c^Disease-specific guidance document(s) in four therapy areas of interest

^d^While this manuscript was under review, the European Member State Coordination Group on Health Technology Assessment (HTA CG) published updated “Guidance on outcomes for joint clinical assessments” (a further development of the previous EUnetHTA guideline), adopted on 10 June 2024

AEMPS, Spanish Agency of Medicines and Medical Devices; AIFA, Agenzia Italiana del Farmaco; C2H, Center for Outcomes Research and Economic Evaluation for Health; CADTH, Canadian Agency for Drugs and Technologies in Health; CIPM, Inter-Ministerial Pricing Commission for Pharmaceuticals; EUnetHTA, European Network for Health Technology Assessment; G-BA, Federal Joint Committee; GENESIS, Grupo de Evaluación de Novedades, Estandarización e Investigación en Selección de Medicamentos; HAS, Haute Autorité de Santé; ICER, Institute for Clinical and Economic Review; IQWiG, Institute for Quality and Efficiency in Health Care; NCMHTA, National Center for Medical and Health Technology Assessment; NHSA, National Healthcare Security Administration; NICE, National Institute for Health and Care Excellence; NSCLC, non-small cell lung cancer; PBAC, Pharmaceutical Benefits Advisory Committee; PED, patient experience data; SLE, systemic lupus erythematosus; UC, ulcerative colitis

The only standalone guidance documents on PED identified (n = 2) were published by the European Network for Health Technology Assessment (EUnetHTA); however, all other HTA agencies in North America, Europe, and Asia Pacific have published general HTA guidance documents including PED-related recommendations (total n = 16), except China. Of note, the China NCMHTA has published one disease-specific guidance document including PED-related recommendations in oncology. The Canadian Agency for Drugs and Technologies in Health (CADTH) has also published disease-specific guidance documents, including PED-related recommendations in oncology and ulcerative colitis.

The level of PED-related guidance issued by HTA agencies varied between countries (Table 5). In some countries, such as Spain, the UK, and Japan, economic evaluation is key to determining the value of a drug; therefore, there were limited PED expectations beyond those required to estimate utilities. In these countries, generic HRQoL instruments are needed because comparability across indications is required, with the EQ-5D often the preferred instrument. Beyond HRQoL, there is little interest in PED on signs, symptoms, or disease impact on functioning. As a result, no or limited guidance on instrument validation, analysis, or interpretation was provided, and no PED guidance on missing data, study design, or study endpoints has been published by the HTA agencies in these countries. In other countries, although economic evaluations are also required, there were some expectations regarding PED validation, analysis, and interpretation (e.g., in France). In countries where COAs are not used to estimate the therapeutic value evaluations are also of new treatments in terms of utilities, more comprehensive details on PED expectations were set out by the HTA agencies. For example, in Germany, the Federal Joint Committee and the Institute for Quality and Efficiency in Health Care (IQWiG) were interested in both generic and disease-specific HRQoL instruments, although no instruments were specified in the guidance documents, as well as PED on signs and symptoms. Furthermore, detailed PED-specific requirements were outlined regarding scale interpretation and handling of missing data. Across all HTA agencies, PED were expected to be collected through COA instruments, and HRQoL was a key concept of interest. Across HTA agencies that discussed PED in more detail, the instruments were expected to be validated in terms of language and culture in the local population, if possible. The interpretation of COA scores varied. While most agencies were interested in MWPC thresholds defined based on anchor analyses, IQWiG considered that the MWPC threshold was at least 15% of the scale range. The French, German, and Australian agencies had specific, but different, rules or recommendations related to missing data. Haute Autorité de Santé (HAS) required that PED analyses were pre-specified to be considered in the HTA. No information about PED beyond COAs was included in any HTA guidance documents; however, some HTA agencies (e.g., CADTH, the National Institute for Health and Care Excellence [NICE], and HAS) sought patient advocacy group input for their assessments, and/or patient representatives were involved in the discussions.

**Table 5:** Overview of Health Technology Assessment Agency/Organization Recommendations Regarding Patient Experience Data by Agency/Organization and Geography/Country

	CADTH	ICER	EUnetHTA	HAS	G-BA & IQWiG	AIFA	CIPM, GENESIS & AEMPS	NICE	PBAC	NHSA & NCMHTA	C2H
Types of PED	“Outcome measure”	COAs	COAs (generic and disease-specific)	COAs (generic and disease-specific)	COAs (generic and disease-specific)	COAs (generic and disease-specific)	COAs (generic)	COAs (generic)	COAs (generic and disease-specific)	PROs	COAs (generic)
Concepts of interest	HRQoL, utilities, QALYs,work productivity, caregiver time, core outcome set	Changes in symptoms/conditions people feel affect quantity or quality of life; HRQoL; QALYs; evLYG	Outcomes related to patients’ feelings, beliefs, preferences, needs and functions; utilities; HRQoL	HRQoL, QALYs	HRQoL, signs, symptoms	HRQoL	HRQoL to derive QALYs	HRQoL to derive QALYs	HRQoL to derive QALYs	Inclusion of PROs in efficacy assessments; HRQoL to derive QALYS	HRQoL to derive QALYs
Recommended instruments/tools	EQ-5D-3/5L, HUI2 or 3, SF-6D. Disease-specific instrument accepted if generic instruments not optimal	No	Use COS when available for specific disease; generic instruments; disease-specific instruments	EQ-5D for utilities, from the age of 16 years. Before the age of 16 years, paediatric systems is recommended	No	Examples provided—EQ-5D-3L, SF-36	EQ-5D (SF-6D and SF-36 accepted)	EQ-5D preferred. If EQ-5D not optimal, validation methods should be described and comparable to those used for EQ-5D Optionally, condition-specific, preference-based measures	MAUI questionnaires (AQoL, CHU9D, EQ-5D-3/5L, HUI2 or 3, SF-6D or SF-36)	HRQoL	EQ-5D-5L (preferred)
COA validation	If disease-specific instrument is used, its measurement properties are considered Language and cultural validation on local population should be considered Copies of validity of outcome measure references available should be provided	–	Use valid, reliable, responsive, and acceptable instruments with documentation Language and cultural validation on local population should be considered	Validated scales appropriately adapted to objective should be used Validated in patient population intended for use	Prerequisite is use of validated instrument and suitable response criterion	HRQoL data should be reported using internationally validated scales	–	Should be established in indication intended for use Peer-reviewed validation study required for novel instruments	No validation information needed for the recommended MAUIs Other instruments must be valid and reliable. Information on responsiveness, interpretation, rationale for use, domains/concepts covered, and scoring methods must be provided	–	Preference-based measures must be validated in Japan When not available, validated PROs should be mapped to preference-based measures
COA analyses/interpretation	Evidence of clinical importance of outcome measure must be provided	–	Responder definition (e.g., PASS or MCID) Switching to different disease stage or severity Graphical representation of the results encouraged Analyses should be pre-specified in protocol and SAP as a primary analysis	Objective and clinical relevance threshold should be pre-specified in protocol MCID threshold should be justified	IQWiG: clinical relevance threshold set to ≥ 15% of scale range for binary outcome For continuous outcomes: predefined thresholds for standardized mean differences GB-A: may accept established MWPC thresholds for consistency reasons where have informed previous HTA decisions	–	–	–	Interested in "responder definition," defined as MWPC threshold, to support COA interpretation MWPC threshold should be based on anchor-based analyses but alternative approaches (statistical or consensus) potentially acceptable Details on anchors are expected	–	–
Missing data^a^	–	–	Follow HRQoL instrument-specific guidelines for handling missing data if available Should be replaced in analysis with value derived from hypotheses about HRQoL of patients with missing data	Missing data should be limited Should describe quantitatively, their random or non-random character, any correction methods used	Critical threshold for missing data: ≥ 30% Critical threshold for treatment arm differences in missing data: 15% Statistics of missing data to be presented for each time point/study visit	–	–	–	Require rationale, mitigation technique, assessment of how similar/different missing patients were to non-missing patients, estimate of whether missing data expected to impact treatment effect, and methods of adjustment for response bias	–	–
Trial design	Designs of clinical trials should be assessed to identify features that may affect use or interpretation of data, e.g., fitness for purpose, credibility, consistency	–	Consider possibility of non-response due to ill health and possible solutions to this risk e.g., alternative modes of administration for HRQoL instrument(s) if risk of higher drop-out rate with self-completed paper questionnaires due to ill health	QoL findings should be based on rigorous methodology, including double-blind conditions, management of multiplicity, appropriate analysis frequency, time, and duration	Open (i.e. non-blinded) studies are of limited validity because of high risk of bias for subjective outcomes. RCTs preferred	–	–	–	–	–	–
Endpoints	In context of RWE, clinical outcome preferred over surrogate At minimum, use COA as secondary outcomes when primary is surrogate	–	Common endpoints include mortality, morbidity, clinical status, symptoms, function or HRQoL Outcome should be long term or final where possible	–	Information on treatment outcomes is based on endpoints relevant to patients (e.g., mortality, symptoms, complication, and HRQoL)	–	–	–	–	–	–

^a^Guidance on acceptable levels of missing data and how best to handle missing data during analyses

AEMPS, Spanish Agency of Medicines and Medical Devices; AIFA, Agenzia Italiana del Farmaco; AQoL, Assessment of Quality of Life; C2H, Center for Outcomes Research and Economic Evaluation for Health; CADTH, Canadian Agency for Drugs and Technologies in Health; CHU9D, Child Health Utility nine dimensions; CIPM, Inter-Ministerial Pricing Commission for Pharmaceuticals; COA, clinical outcome assessment; COS, core outcomes set, EUnetHTA, European Network for Health Technology Assessment; evLYG, equal value life years gained; G-BA, Federal Joint Committee; GENESIS, Grupo de Evaluación de Novedades, Estandarización e Investigación en Selección de Medicamentos; HAS, Haute Autorité de Santé; HRQoL, health-related quality of life; HUI2 or 3, Health Utilities Index Mark 2 or Mark 3; HTA, health technology assessment; ICER, Institute for Clinical and Economic Review; IQWiG, Institute for Quality and Efficiency in Health Care; MAUI, multiple-attribute utility instrument; MCID, minimal clinically important difference; MWPC, meaningful within-patient change; NCMHTA, National Center for Medical and Health Technology Assessment; NHSA, National Healthcare Security Administration; NICE, National Institute for Health and Care Excellence; PASS, patient acceptable symptom state; PBAC, Pharmaceutical Benefits Advisory Committee; PED, patient experience data; PRO, patient-reported outcome; QALY, quality-adjusted life year; QoL, quality of life; RCT, randomized controlled trial; RWE, real-world evidence; SAP, statistical analysis plan; SF-36, 36-item Short Form Survey; SF-6D, Six-dimension Short Form Survey

## Discussion

We have described guidance relating to PED issued by regulatory and HTA agencies in North America, Europe, and Asia Pacific. Overall, our findings indicate that the voice of patients by means of PED plays an increasingly important role in regulatory decision-making and HTA, with the US FDA, EMA, China NMPA-CDE, and EUnetHTA having published guidance specifically on the use of PED in clinical trials. The majority of identified regulatory and HTA guidance documents were published within the last 5 years (2019–2023), indicating that the PED environment is changing rapidly.

### Regulatory Guidance

The FDA is leading the way in advancing PED measurement standards, and regulatory agencies in Europe (and by extension Australia), China, and Japan tend to be consistent with the FDA’s approach, although they generally offer less detailed guidance. It is evident that regulatory agencies are aware of what is being done by other agencies around the world, and interact with other agencies, resulting in a trend towards global regulatory harmonization through the adoption of common PED measurement standards. For example, Australia’s TGA adopted EMA guidance on the use of PRO measures in oncology studies [27, 29], and FDA guidance on real-world evidence [76, 77]. Furthermore, guidance documents published by the NMPA-CDE in China [30–32] and the PMDA in Japan [34] mention FDA and EMA guidance. In addition, like the FDA, the EMA contributes to the development of global guidance (e.g., with the Council for International Organizations of Medical Sciences, and the International Council for Harmonisation of Technical Requirements for Pharmaceuticals for Human Use [ICH]), and regularly engages in discussions with other decision-makers and medicines developers [78]. Finally, the EMA’s Regulatory Science Strategy to 2025 “recognizes the need to identify optimal approaches for engaging patients in medicines development and benefit–risk assessments, including the development of standards for designing, conducting, analyzing, and reporting relevant studies incorporating PED for regulatory submission, and to elucidate how such data can best inform regulatory decisions” [79].

Although no specific guidance on the use of PED has been issued by the Canadian or English regulatory agencies, both agencies are working on ongoing PED-related initiatives. In 2019, the Canadian PROs National Steering Committee drafted an oncology-focused national position statement with the intention of transforming the healthcare system by supporting the integration and implementation of PROs into the Canadian healthcare setting and by advancing PRO knowledge through research [80]. The first published Canadian national position statement on PROs was intended as a step toward the national alignment of stakeholders (HCPs, administrators, health policymakers, patient advocacy groups, researchers, and industry) who support the greater use of PROs in Canadian healthcare and research contexts, outlining recommendations in four main areas: patient and family, health policy, clinical implementation, and research. In 2021, having undertaken additional responsibilities in the UK post-Brexit, the MHRA announced a broad new “Patient Involvement Strategy 2021–2025” outlining five key patient-focused objectives for the agency: patient and public involvement, responsiveness, internal culture, measuring outcomes, and partnership [81]. Although the MHRA has not yet provided any specific guidance on PED, this initiative appears to place a value on PED, recommending “developing the use of PROs so that it is built into all our licensing decisions” and monitoring the “number of clinical trial protocols for which the agency recommended that PRO measures should be built into their design” [81]. Furthermore, a more recent strategy publication described the need to develop new guidelines and processes to expand patient engagement safely and ethically as a priority [82].

### HTA Guidance

Interest in PED and the level of expectations regarding PED vary considerably across HTA agencies in North America, Europe, and Asia Pacific. In countries where economic evaluation is key to determining the value of a drug, such as Canada and the UK, guidance on expected methods for the use of PED to estimate utility values is detailed, but recommendations beyond this use are limited. Although agencies such as NICE do not appear to put a lot of emphasis on PED, they are engaging with patients through alternative channels. Patients sit at the negotiating table, and agencies engage with patient advocacy groups to seek their input. However, although patient engagement activities do not exclude the use of PED collected through research, or vice versa, these two approaches are likely to provide different types of information on different topics. Interestingly, a separate global review of the patient engagement and PED landscape also demonstrated increasing interest and guidance on their use individually in decision-making, but highlighted the lack of guidance on maximizing the value of patient input into decisions by combining patient engagement and PED into both regulatory and HTA processes, suggesting that the necessity of integrating patient engagement in the design and interpretation of PED programs should be highlighted [83].

National HTA agencies interested in the use of COAs beyond the estimation of utility values, such as those capturing HRQoL and disease symptoms, have issued PED-related guidance with considerable variation in the level of detail. In countries such as France and Germany—where HTA agencies give more weight to COAs along with more detailed guidance—the approach is generally consistent with that of the FDA, despite some nuances, such as the expected responder threshold. However, in the Asia Pacific region, country-specific guidance is much more varied.

Notably, across North America, Europe, and Asia Pacific, where HTA agencies have expressed interest in PED derived from a non-COA source, the guidance is consistently limited.

Overall, our results are consistent with those of a previous review of oncology HTA evidence submission requirements across Europe, which found that differences exist between HTA agencies regarding guidance on how PRO data should be collected, reported, and analyzed, as well as how the data are reviewed and considered in oncology assessments [84].

### Alignment Between HTA and Regulatory Expectations

Globally, there is generally alignment between regulatory and HTA agencies about PED expectations when HTAs do not focus solely on utilities. Therefore, if US FDA guidance is closely followed when initiating a development program, the evidence should meet most expectations of other regulatory and HTA agencies around the world.

In general, instruments able to assess utilities for HTA purposes (i.e., generic multi-attribute instruments) should be included in international development programs in addition to instruments suitable for assessing concepts relevant for the regulatory agencies. Therefore, it is important to recognize these differences in concepts of interest between regulatory and HTA agencies, which should perhaps be expected given that the goals of these two types of agencies are different, early in the drug development process. Regulatory agencies conduct risk–benefit assessments of the efficacy and safety of new drugs and, as such, are not interested in utilities but value specific disease information, whereas HTA agencies generally either conduct incremental cost–benefit assessments versus the current standard of care (i.e., cost-effectiveness analyses based on utilities), or assess the overall value of new medicines, based on factors such as HRQoL data or effectiveness.

The other differences identified between regulatory and HTA agencies concern the interpretation of PED (e.g., what is a meaningful difference) and the cultural validation of instruments in Europe and the Asia Pacific region.

One downside of the misalignment between various HTA bodies and the differing requirements of HTAs and regulators is that study protocols are becoming increasingly complicated, adding to the burden of bringing new drugs to market. This misalignment can necessitate the collection of various types of PED to meet all HTA and regulatory requirements, resulting in significant patient burden and potentially differences in data interpretation or gaps in the expected data. Greater alignment among bodies could alleviate this issue.

In this regard, collaborative regulatory and HTA agencies’ initiatives to improve evidentiary alignment between stakeholders are vital to improve the quality and appropriateness of data generated by drug developers in view of future HTAs [85–88]. For example, the parallel EMA/EUnetHTA 21 Joint Scientific Consultations offer non-binding scientific advice before the start of pivotal clinical trials [86]. Finally, the ICH has guidance in development and currently under public review (as of April 2024) which is aimed to support harmonization of methodologies, standards, and data requirements and integration of data for regulatory submissions; this guidance should be considered in future reviews once it is available in its final form which is projected to be in late 2026 [89].

### Call for Action

Although PED is important now and will be more important in the future, in that it has the potential to inform regulators and health technology assessors on key unmet needs, endpoints, benefits, and risks that matter most to patients and their acceptable trade-offs [90], some countries are more advanced in their use of PED than others. However, there is a trend towards PED initiatives (e.g., published workshop minutes, or discussion documents) among the countries that are less advanced in their use of PED, suggesting efforts are being made to narrow the gap by those agencies currently lagging behind the leaders in PED.

In terms of harmonization, agencies that are catching up should try to align with existing PED measurement standards or adopt existing guidance, instead of developing their own guidance. Exchange and dialogue between HTA and regulatory agencies that have already produced or are producing guidance may identify areas amenable to harmonization and thus further facilitate patients’ access to beneficial drugs. The EUnetHTA/EMA Joint Scientific Consultations provide an ideal platform to enable this type of collaboration in Europe [86]; however, it should be acknowledged that there may be cultural or regional differences that might also have an impact on PED and the way it is analyzed, limiting the direct transferability of PED results. Furthermore, differing payment models across countries may affect HTA requirements.

In addition, there is a need to go beyond COA (e.g., to preference studies, qualitative research, and/or natural history examinations) especially in rare diseases. Minutes from a recent EMA decision-making workshop on PED in European medicines development highlighted that “PED does not only involve quantitative sources of evidence (e.g., patient-reported outcomes or patient-reported experience measures) but also qualitative sources (i.e., any information obtained as part of patient engagement activities that reflect the wider perspective of patients’ experience, for example, the outcome of focus groups, surveys or interviews)” [6]. However, these methods of generating PED have yet to be included in published guidance documents.

Despite these challenges, having a global picture of international PED-related requirements could support the development of a checklist to guide the efficient, standardized collection and presentation of PED, and support the effective review of PED by both regulatory and HTA agencies. Specifically, the ideal checklist would meet international regulatory and HTA standards regarding COA collection, validation, implementation, analyses, interpretation, and reporting to enable applicants to improve the quality and clarity of reported information, guide assessors in the review of COA data, and further support evidence-based decision-making. Such a checklist would also help patient representatives, HCPs, research groups, sponsors, and regulatory and health technology assessors to better understand presented COA data, evaluate its quality, and allow for comparative research and synergies between stakeholders. The patient perspective must be consistently incorporated into research, and the research community should provide information on how various treatment options might affect important patient outcomes to patients, family members, and HCPs.

### Limitations

We selected only 7 regulatory agencies and 12 HTA agencies for inclusion in this analysis; therefore, our findings may not be generalizable to all agencies and geographies globally. However, considering the depth of the analysis conducted, the inclusion of 22 agencies was considered sufficient to understand the nuances between regulatory and HTA guidance, and between individual agency guidance documents. The focused objectives and search terms in this study aimed to identify the depth of guidance and process descriptions relating specifically to how PED should be included in regulatory or HTA submissions and what types of evidence are expected when they are included, which may have limited the breadth of the documents and guidance available. Future studies might consider broader objectives to provide additional insights into the availability of guidance and expectations of the use of PED with an expanded list of search terms such as those included in the FDA’s PED definitions, for example, “preferences for treatment”, “benefits and risks”, “patient engagement”, and “patient involvement.” In particular, the exclusion of documents describing patient engagement activities may have limited the breadth of information gathered, including insights that may have affected the study’s conclusions. Additionally, future studies may also consider an assessment of the available guidance relating specifically to medical devices.

The objectives of this study were focused on identifying evidentiary requirements for PED, as well as procedural steps for including and submitting PED to regulatory agencies and HTA bodies. Given this technical focus, expertise within the study team related to the collection and use of PED in regulatory/HTA processes was leveraged in the study design, search strategy, document review and extraction parameters, analysis and reporting. It was not our objective to interpret or comment on how additional engagement with or insights from patients should be incorporated into PED guidance; therefore, patient partners were not included in this study. However, we recognize the highly valuable contributions that patient partners can make in the identification, interpretation, and reporting of research, and that this could be considered a limitation in the current study. We recommend that future studies with a different focus consider the inclusion of patients as partners throughout the research process (from study design through to reporting and publication) to leverage the value they have to offer in ensuring relevance, clarity, and interpretation.

Furthermore, although it is recognized that there may be a gap between recommendations in published guidance and actual assessment processes, our analysis did not investigate how PED were valued during dossier reviews. For example, HTA agencies may overlook COA data and/or have low confidence in its quality and value. Alternatively, different HTA agency reviews may result in different recommendations, potentially due to contextual differences between geographies. Further searches and analyses are needed to explore this question. However, the issuance of numerous PED-related documents over the past 5 years suggests that PED is considered important. The value of PED may continue to grow as guidance recommendations are integrated into research and reviews.

## Conclusion

In this study, we aimed to identify and describe guidance relating to PED issued by regulatory and HTA agencies in North America, Europe, and Asia Pacific. The search strategy used several relevant key terms to ensure adequate data capture and focused on four therapeutic areas of interest. A detailed review of each identified guidance document was undertaken to examine information related to types of PED evidence required or expected by regulatory/HTA agencies, concepts of interest, instruments/tools, COA analysis, interpretation and evaluation, and study design elements including endpoints. Using this process, we identified 34 regulatory and 21 HTA documents, most of them being published in the last 5 years.

From our analysis, we conclude that, through PED, patient voice is increasingly being recognized as an important contributory factor to regulatory decisions on market authorization, access, and reimbursement decision-making via the HTA process. Therefore, the collection of the PED required by regulatory and HTA agencies should be incorporated early in drug development and evidence-generation plans. The FDA is currently leading the way in advancing PED measurement standards and expectations. Despite some discrepancies (for example regarding the interpretation of PED), there is generally alignment between regulatory and HTA agencies about PED expectations when HTAs do not focus solely on utilities; whereas, in countries where economic evaluation is key to determining the value of a drug, recommendations beyond the use of utilities are limited. Misalignment can necessitate the collection of multiple types of PED to meet all requirements and lead to differences in data interpretation or gaps in the expected data. This is why harmonization could be helpful. Regulatory and HTA agencies considering PED-related initiatives in the context of international clinical trials should think about aligning their approach with other stakeholders in the drug development process. A culture of PED use is lacking in some countries, with the added value of PED not being recognized. Further education on the culture of PED is needed to demonstrate the value of evidence coming from the PED perspective and effect a change in mindset in some countries. Harmonization of PED measurement standards is the target, ensuring that decisions can be made based on valid and reliable PED, while acknowledging that decisions may ultimately vary given different national needs, priorities, and biases.

## Data Availability

All reported data are available in the public domain.
